# Abscess of the Liver Treated by Puncture

**Published:** 1847-10

**Authors:** 


*Abscess of the Liver treated by Puncture.*—The following cases reported in the *Medical Times* by Dr. Clay, is sufficiently rare in this country to deserve further publicity:—

The patient complained of fixed pain in the right superior portion of the umbilical region, for which he was treated antiphlogistically without relief. His bowels were constipated; countenance yellow; spirits depressed; anorexia; pulse 90; evident enlargement of the liver, with paucity of bile. He took ox-gall, dr. ij.; calomel, gr. x., divided into twenty-two pills, of which, one three times a day was the dose. Under this plan he quickly improved, and remained well until after bathing, when the fixed pain returned. Being at this time in a different locality, he was again treated by bleeding, &c., and as before without benefit. He then took the ox gall and calomel, and a second time became greatly relieved. Dr. Clay lost sight of him from this time, but it appears that while in Dublin he suffered a severe relapse, with pain in the old spot, which had become more tense and permanent. At this spot Dr. Clay passed a grooved needle, and as it gave issue to a drop of pus, he tapped it freely with a trocar, and drew off four pounds of fœtid pus. At each dressing for several days a pound of pus escaped, but after that time the discharge gradually diminished, and at the end of three months the man was completely recovered. Dr. Clay calculated that in all, at least sixteen pints of matter must have been discharged. The treatment after the evacuation of the abscess was tonic and alterative, the functions of the liver being restored by the ox gall and calomel.—*Monthly Journal.*

